# Association of the Receptor for Advanced Glycation End Products Gene Polymorphisms and Circulating RAGE Levels with Diabetic Retinopathy in the Chinese Population

**DOI:** 10.1155/2013/264579

**Published:** 2013-11-04

**Authors:** Li Yang, Qunhong Wu, Yuan Li, Xiaohong Fan, Yanhua Hao, Hong Sun, Yu Cui, Liyuan Han

**Affiliations:** ^1^The 2nd Affiliated Hospital of Harbin Medical University, Department of Social Medicine, School of Public Health, Harbin Medical University, Harbin, Heilongjiang 150086, China; ^2^Department of Social Medicine, School of Public Health, Harbin Medical University, 157 Baojian Road, Harbin, Heilongjiang 150081, China; ^3^Department of Preventive Medicine, Medical School of Ningbo University, Ningbo, Zhejiang 315211, China

## Abstract

*Objectives*. This study investigated the association between polymorphisms in the receptor for advanced glycation end products (RAGE) gene and the susceptibility to diabetic retinopathy (DR) in a Chinese population and identified a correlation between serum-soluble RAGE (sRAGE) levels and DR risk. *Materials and Methods*. We enrolled 1040 patients with type 2 diabetes mellitus: 372 patients with DR and 668 without retinopathy (NDR). All polymorphisms were genotyped by time-of-flight mass spectrometry. Serum levels of sRAGE were assayed by enzyme-linked immunosorbent assays. The interaction of SNPs was analyzed by multifactor dimensionality reduction (MDR). *Results*. The frequency of the SS genotype for the G82S polymorphism was 12.4% in the DR group and 6.6% in the NDR group; this difference was significant. G82S was associated with sRAGE levels. Specifically, after adjustments for age, sex, duration, and glucose metabolism, serum sRAGE levels were significantly higher in DR subjects with the S/S genotype than in NDR subjects in general. In the DR group, subjects with the G/S genotype had lower sRAGE levels than subjects with the G/G or S/S genotype (P < 0.01). The best multilocus genetic interaction model was assessed using the MDR method; 2184A/G, 1704G/T, G82S, and −429T/C were identified. *Conclusions*. The findings suggest that the G82S polymorphism in the *RAGE* gene is associated with DR risk, and G82S was associated with circulating levels of sRAGE. The mechanism by which G82S polymorphism modulates the sRAGE levels remains to be elucidated.

## 1. Introduction

The incidence of diabetes is rapidly increasing in developing countries, especially in China. According to the latest report from the Chinese Diabetes Society (CDS), the prevalence of diabetes rose to 9.7% in 2010 in China; approximately 92.4 million adults (age ≥ 20 years) are affected. 

Diabetic retinopathy (DR) is a potentially devastating microvascular complication in diabetes and one of the leading causes of vision loss and blindness worldwide. DR occurs in approximately 35.7% of patients with diabetes in China [1]. The pathogenesis of DR is complex, but severe hyperglycemia is the major risk factor for developing retinopathy [2]. Prolonged hyperglycemia is required for the development of anatomic retinal vascular lesions in most animal models and in humans with DR [3]. Chronic exposure of the retina to hyperglycemia leads to the formation and accumulation of advanced glycation end products (AGEs), which play an important role in the progression of retinopathy.

AGEs are proteins or lipids that become nonenzymatically glycated after exposure to sugars. Prolonged hyperglycemia, dyslipidemia, and oxidative stress in diabetes increase the production and accumulation of AGEs in the diabetic vasculature [4]. The effects of AGEs are partially mediated by cellular receptors, the most important being the receptor for AGE (RAGE). RAGE is a member of the immunoglobulin superfamily of cell surface molecules and is expressed ubiquitously in various retinal cells. In animal models and humans with diabetes, RAGE expression in the retina increases, concomitant with AGE accumulation [5, 6]. Upregulation of RAGE in the retinas of patients with diabetes activates prooxidant and proinflammatory signaling pathways [7]. The AGE-RAGE interaction activates multiple intracellular signaling pathways and subsequently evokes oxidative stress and inflammatory responses in vascular cells, resulting in the transcription of target genes and oxidative stress, thus contributing to the development and progression of DR [8].

RAGE has a C-terminal-truncated secretory isoform, termed soluble RAGE (sRAGE), which neutralizes AGE-mediated damage by acting as a decoy [9]. In humans, sRAGE is produced by alternative splicing of RAGE messenger RNA or by proteolytic cleavage and shedding of cell-bound RAGE [7]. High levels of sRAGE are present in the circulatory system. Given its AGE-binding properties, sRAGE might play an antagonistic role by competing with cell surface RAGE, thus preventing the RAGE-mediated inflammatory response. As such, the proportion and production of sRAGE may influence RAGE-mediated functions in various tissues and inflammatory conditions [10, 11].

The *RAGE* gene, composed of a 1.7-kb 5′ flanking region and 11 exons [12], is located on chromosome 6p21.3 in the MHC locus. It is an attractive candidate gene for influencing DR. Sequence variation within the *RAGE* gene has been studied, and a relatively large number of single nucleotide polymorphisms (SNPs) in the *RAGE* coding and noncoding regions have been identified [13, 14], including the exon polymorphism G82S, the promoter region polymorphism −429T/C, and several intron polymorphisms (1704G/T, 2184A/G, and rs1035798). Many of the findings, however, are inconsistent. 

In this study, we investigated the higher-order interactions among the abovementioned polymorphisms as they relate to DR risk. We performed a cross-sectional study to determine (1) the relationship between genetic polymorphisms in the *RAGE* gene (2184A/G (rs3134940), 1704G/T (rs184003), rs1035798, G82S (rs2070600), and −429T/C (rs1800625)) and the risk of DR; (2) potential multilocus interactions that affect the risk of DR; (3) the association between RAGE polymorphisms and sRAGE levels; and (4) the association between sRAGE levels and DR. 

## 2. Materials and Methods

### 2.1. Study Population

This study enrolled 1040 patients diagnosed with type 2 diabetes mellitus based on clinical features, laboratory data, and the guidelines in the recent Expert Committee Report of the American Diabetes Association [15]. Additionally, all participants underwent a fundus fluorescein angiography by certified ophthalmologists. Three-hundred and seventy-two patients were diagnosed with DR as a complication of diabetes (DR group), and 668 diabetic patients without DR were included in the study for comparison (NDR group). The following were excluded: (1) patients with diabetes undergoing thiazolidinedione therapy; (2) patients with hypertension undergoing angiotensin-converting enzyme inhibitors therapy; (3) patients with diagnosed diabetic nephropathy; (4) patients with acute or chronic inflammatory disease; and (5) patients with type 1 diabetes, maturity-onset diabetes of the young, or mitochondrial diabetes. All participants provided written informed consent. The protocol for this study complies with the Helsinki declaration. It was approved by the Committees on the Ethics of Human Research of Harbin Medical University. 

Clinical data were recorded with respect to age, sex, body mass index (BMI), diabetes duration, systolic blood pressure, diastolic blood pressure, waist-hip ratio (WHR), total cholesterol (TC) level, triglyceride (TG) level, high density lipoprotein-cholesterol (HDL-C) level, low density lipoprotein-cholesterol (LDL-C) level, fasting plasma glucose (FPG) level, 2 h postprandial plasma glucose (2hPG) level, and hemoglobin A1c (HbA1c) level. 

### 2.2. Gene Variants and Genotyping

The selection of particular SNPs was based on the following: (1) a population frequency, based on the minor allele frequency (MAF), of >5%; (2) known functional or regulatory impact; and/or (3) previously described association with DR.

Genomic DNA from peripheral blood leukocytes was extracted from 200 *μ*L of whole blood obtained at the time of the interview, using the TIANamp Blood DNA Kit (TIANGEN, China) according to the manufacturer's instructions. The DR and NDR groups were mixed for genotyping, and laboratory personnel were blinded to the patient's diagnosis (DR versus NDR). 

Genomic DNA was diluted to a final concentration of 15–20 ng/*μ*L for the genotyping assays. Genotyping for each participant was performed using a MassARRAY compact analyzer based on the chip-based matrix-assisted laser desorption ionization time-of-flight mass spectrometry platform (Sequenom, San Diego, CA, USA). The process was as follows: (1) DNA isolation, (2) primer design, (3) PCR, (4) neutralization of unincorporated dNTPs (SAP Reaction), (5) extension reaction, (6) conditioning of iPLEX Gold reaction products, (7) application to the SpectroCHIP II array, (8) definition of assays and plates, and (9) spectrum acquisition and analysis. Primers for the polymerase chain reaction and single base extension were designed using SEQUENOM Human Genotyping Tools (online) and SEQUENOM MassARRAY Assay Design v4.0 software. 

### 2.3. Biochemical Analysis

Blood samples were obtained after an overnight fast from each participant for baseline measurements, and the samples were stored at −80°C until use. Plasma glucose levels, 2hPG levels, total cholesterol levels, HDL levels, and triglyceride levels were also measured using enzymatic methods. LDL levels were calculated using the Friedewald equation. HbA1c levels were assayed using high-performance liquid chromatography with a cation exchange column.

### 2.4. ELISA for sRAGE

sRAGE was measured by sandwich colorimetric ELISA using commercially available kits (human sRAGE ELISA kit; R&D Systems, Abingdon, UK) with a standard range of 31.25–2000 pg/mL and intra-assay and inter-assay coefficients of variation (CV) of <10% and <15%, respectively. The results were expressed in pg/mL.

### 2.5. Statistical Methods

Statistical analysis was performed using the SPSS software package (SPSS 13.0, Beijing, China). Continuous variables were expressed as the mean ± SD. Categorical variables were presented as frequencies. Group differences were analyzed by Student's *t*-test, the Mann-Whitney *U* test, and the Chi-square test for normally distributed, abnormally distributed, and noncontiguous variables, respectively. We performed 3 independent tests for sRAGE levels in individuals with G82S genotypes. To avoid type I error induced by multiple tests, Bonferroni's adjustment for multiple comparisons was applied, and a *P* value of <0.017 was used as the significance threshold.

For genotypic and allelic frequencies, the Hardy-Weinberg equilibrium was applied using SNPStats online tools (http://bioinfo.iconcologia.net/snpstats/start.htm). The linkage disequilibrium (LD) between the RAGE SNPs was assessed with the Haploview software package, and Lewontin's disequilibrium coefficient (*D*′) was estimated.

Univariate analysis and multiple logistic regression analysis were performed for independent determinants of serum sRAGE levels and DR. Multifactor dimensionality reduction version 1.2.5 was used to detect potential multilocus genetic interactions (http://linux.softpedia.com/progDownload/MDR-Download-35200.html). Statistical power was calculated using the software PASS (http://www.ncss.com/). A *P* value of <0.05 was considered statistically significant for all analyses.

## 3. Results

### 3.1. Power Analysis

Power analysis was performed to determine the number of subjects needed for this study. Using the equation of contingency table, we achieved values of 99% for the Chi-square test and determined an appropriate sample size of 311 subjects. For this study, 1040 subjects were included, more than required to detect statistical significance.

### 3.2. Clinical Characteristics of the Groups

The basic characteristics of the 2 groups are shown in Table 1. Each group was significantly different with respect to duration and the levels of FPG, 2hPG, HbA1c, and LDL-C. 

### 3.3. Polymorphism Distribution in the DR and NDR Groups

The genotype and the allele distributions of the 5 RAGE polymorphisms analyzed in this study are shown in Table 2. The genotype frequencies of the 2 groups are in accordance with those predicted by the Hardy-Weinberg equilibrium for all polymorphisms (except for rs1035798 in DR group). Linkage disequilibrium among the studied polymorphisms was not detected. A significant association was observed between the SS genotype and the risk of DR; the other polymorphisms showed no statistically significant association with DR.

### 3.4. Multilocus Genetic Interactions and the Risk of DR

**Figure 1:**
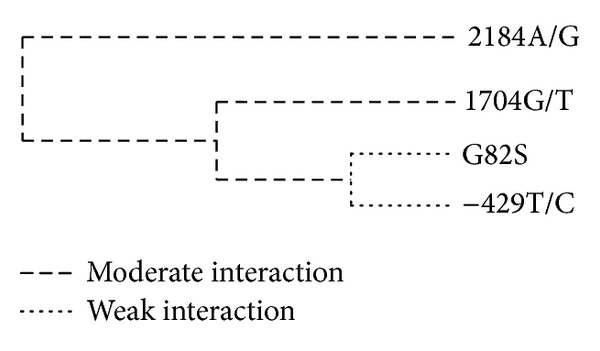
Interaction dendrogram for the best 4-locus SNP model selected by the MDR. The dashed line indicates moderate interaction, and the dotted line indicates independence.

Multilocus genetic interactions were analyzed by MDR. Each genotype was categorized into 2 subgroups, a high-risk group and a low-risk group, according to the ratio of cases to controls with the genotype. The prediction accuracy of the model was estimated by tenfold cross-validation. The best model chosen had the highest cross-validation consistency (CVC) and the highest prediction accuracy. CVC, training balance accuracy, testing balance accuracy, associated *P* values, OR, and 95% CI were obtained from the MDR analysis. The best model of multilocus analysis is shown in Table 3. One 4-locus model had a maximum CVC of 10 and a maximum prediction accuracy of 54.92%. The best model consisted of 4 SNPs (2184A/G, 1704G/T, G82S, and −429T/C).  Figure 1 shows an interaction dendrogram highlighting the information obtained using MDR. A dashed line indicates moderate interaction, and a dotted line represents independence. The interaction dendrogram indicated that this set, which included the 2184A/G and 1704G/T polymorphisms, exhibited a moderate synergistic effect, whereas G82S and −429T/C had no synergistic effect. 

### 3.5. Relation between RAGE G82S Genotypes and sRAGE Levels

**Figure 2:**
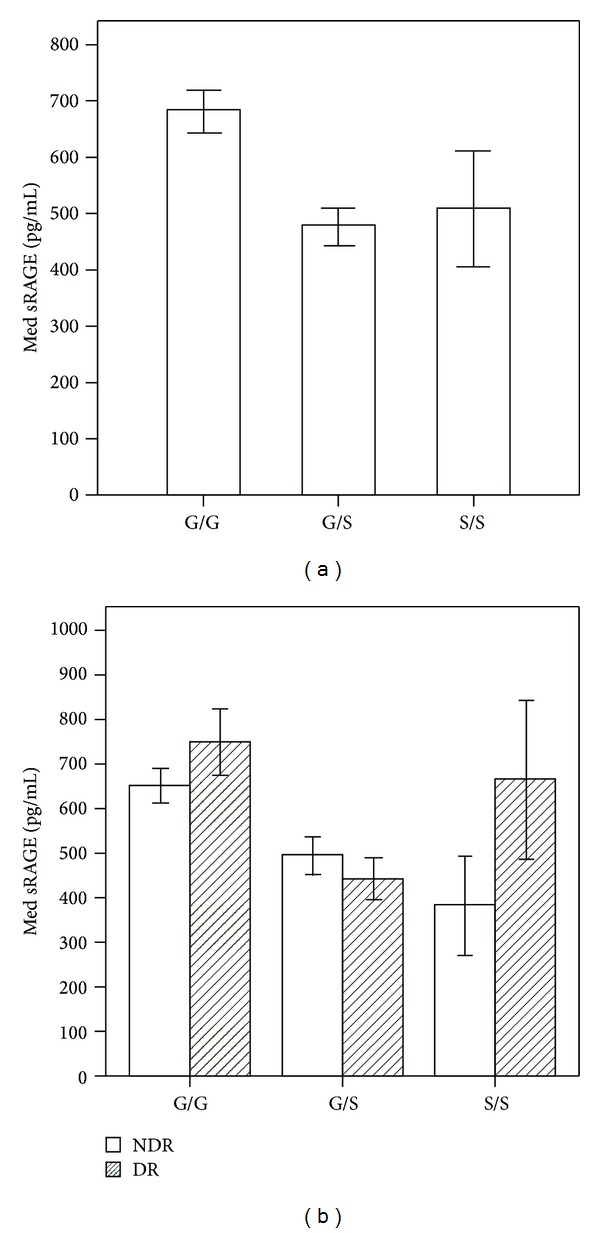
Influence of RAGE G82S genotypes on sRAGE. All diabetic subjects with the G/G genotype had significantly higher sRAGE concentrations than those with G/S genotype and S/S genotype (bonferroni adjustment *P* < 0.01, (a)). sRAGE concentrations in DR subjects with the S/S genotype were higher than in NDR subjects with the S/S genotype (*P* < 0.05). DR subjects with the G/S genotype had lower sRAGE levels than DR subjects with the G/G and S/S genotypes after adjusting for the duration of diabetes, FPG, 2hPG, LDL, and HbA1c (Bonferroni adjustment *P* < 0.01). Note: error bars: 95% CI.

Because sRAGE levels were nonnormally distributed, we conducted the analysis using a nonparametric test. There was no statistically significant difference between the DR and NDR groups in terms of sRAGE concentration in plasma after adjusting for the duration of diabetes, FPG, 2hPG, LDL, and HbA1c (*P* > 0.05). However, there was a significant association between the G82S genotypes and plasma sRAGE concentrations in all subjects with diabetes (*P* < 0.01, Figure 2(a)). Serum sRAGE levels were significantly higher in DR subjects with the S/S genotype than in NDR subjects with the S/S genotype. In addition, in the DR group, subjects with the G/S genotype had lower sRAGE levels than those with the G/G or S/S genotype. Besides, sRAGE concentrations in NDR subjects with the G/S genotype were significantly higher than in those with the S/S genotype and significantly lower than in those with the G/G genotype (Figure 2(b)).

## 4. Discussion

In this study, the G82S polymorphism in *RAGE* was associated with DR risk. However, we did not find a significant association between other *RAGE* SNPs and DR susceptibility. To our knowledge, this is the first study to investigate the association between several *RAGE* polymorphisms and DR in Chinese populations. Furthermore, we assayed serum sRAGE levels in the DR and NDR groups to better understand the nature of this correlation.

The G82S polymorphism, one of the first polymorphisms identified in the *RAGE* gene, is potentially interesting because it occurs at a predicted N-linked glycosylation in the AGE binding site and thereby influences AGE-RAGE interactions [16]. Several studies have investigated the association between G82S and DR in various populations. However, the results were inconsistent. In our study, the G82S polymorphism was associated with DR, and the SS genotype was a risk factor for DR. This suggests that the G82S SNP in the *RAGE* gene might contribute to DR pathogenesis. Our results are similar to those of another study, which found that the 82S allelic variant increased the risk of DR [17]. Other studies did not find a significant association between G82S and DR [18–20]; however, one did not report the sample size of DR subjects, and the sample size in the other studies was smaller than in this study. Different population histories, which could alter haplotype block structure resulting in noncausative allelic association, or differing genetic and environmental contributions of disease risk in various race and geographic groups might also explain the different results [21]. Additionally, the type of diabetes, duration of diabetes, and other characteristics, such as the patients selected and the sample size, might vary between the studies and influence the results.

In this study, there was no significant difference in the serum levels of sRAGE between the DR and NDR groups. This finding differs from previous studies, in which an association between circulating concentrations of sRAGE and DR was demonstrated. Specifically, lower levels of sRAGE were found in patients who developed DR [22, 23]. Another study demonstrated that sRAGE serum levels were significantly higher in patients who had diabetes and proliferative DR than in patients who had diabetes but no retinopathy [24]. Differences in the subject population (age and duration of diabetes) and ethnicity could account for these discrepancies. However, the sample size in the previous studies was less than 50. Therefore, future prospective cohort studies with large sample sizes are necessary to clarify the relationships between serum sRAGE levels and DR.

Although the association between G82S and circulating sRAGE levels has been previously demonstrated in nondiabetic Korean populations as well as diabetic Caucasian populations [25, 26], this is the first study to confirm these findings in DR populations. In this study, the G82S polymorphism correlated with serum sRAGE in all the patients who had diabetes. In addition, S/S genotype was a risk factor for DR, and DR subjects with the S/S genotype had significantly higher levels of sRAGE than the NDR subjects in general, which suggests the significance increase in the level of sRAGE in DR subjects with the S/S genotype might be associated with an increased risk for developing of DR. Although the mechanism that S/S genotype influences the serum levels of sRAGE is still not understood, our hypothesis is supported by the following observations. (1) RAGE 82S allele upregulates the inflammatory response and may contribute to enhanced proinflammatory mechanisms in inflammatory diseases [27]. (2) RAGE expression was enhanced in diabetic vasculature and sRAGE could be generated from the cleavage of cell surface RAGE extracellular domain in endothelial cells by proteolytic means [28]. sRAGE can be formed by cleavage from the cell-surface receptor isoform [29, 30]. 

 In addition, DR subjects with the G/S genotype had significantly lower serum sRAGE levels than those with the G/G or S/S genotype. In our study, we used the R&D immunoassay to assay total circulating sRAGE. The low sRAGE levels assayed in the G/S genotypes (with serine at location 82) could reflect a higher binding affinity of RAGE for ligand [31] and, consequently, a diminished ability of the R&D antibody to capture sRAGE. The mechanism by which G82S affects the serum levels of sRAGE is unknown, but the polymorphism is thought to have functional significance because it is located in the ligand-binding V domain of RAGE. As such, it may alter the N-linked glycosylation state of the protein, resulting in conformational changes in the protein that influence the detection of sRAGE by the R&D antibody. Furthermore, the polymorphism may influence intracellular processes, resulting in increased activation of proinflammatory proteins such as nuclear factor-*κ*B (NF-*κ*B). In turn, this intracellular signaling process may enhance RAGE expression in the retina through a feedback mechanism that would ultimately affect sRAGE concentrations [27]. 

It is becoming increasingly evident that single-locus effects cannot explain multifactorial chronic diseases. Thus, when the single polymorphism effect is not present alone or is not strong enough, it is important to characterize genes related to susceptibility, keeping in mind the concept of multilocus genetic interactions. Multifactor dimensionality reduction (MDR) is used to analyze gene-environment interactions, and it has been used in many genetics studies of common diseases. To the best of our knowledge, this is the first study to explore the multilocus interactions associated with DR in the Chinese population. Using MDR analysis, we determined that the 4-locus model is the best model to identify DR susceptibility. This conclusion is based on the model's balanced accuracy and CVC. This model included 2184A/G, 1704G/T, G82S, and −429T/C. The interaction dendrogram revealed that the 2184A/G and 1704G/T polymorphisms exhibit a moderate synergistic effect. We did not find an association between 2184A/G and 1704G/T intron polymorphisms and DR. Moreover, Kanková et al. previously established that these 2 polymorphisms might influence oxidative stress [32]. They also found that the 1704G/T and 2184A/G polymorphisms exhibit a similar synergistic tendency in patients with noninsulin-dependent diabetes mellitus and in patients without diabetes. These 2 polymorphisms, located at introns 7 and 8, respectively, may have similar functions and act synergistically. 

The advantages of this study are as follows. First, the cross-sectional design provided a sufficient sample size. The power values of each analysis were more than 90%. Second, this is the first comprehensive study to investigate the correlation between several *RAGE* gene polymorphisms and DR. Third, we examined serum levels of sRAGE in Chinese population. Despite these advantages, the present study has several limitations. The system utilized to detect sRAGE cannot distinguish between sRAGE splice variants. Because the proportion of sRAGE isoforms could differ among individuals, the development of new specific assays will improve the evaluation of sRAGE levels [33]. Additionally, because the number of proliferative retinopathy (PDR) patients was small, we did not further classify DR subjects into nonproliferative retinopathy (NPDR) and proliferative retinopathy (PDR) groups.

Little is known about the mechanisms of sRAGE production and formation. The *RAGE* gene may participate in the regulation of sRAGE expression *in vivo*. Further studies are needed to explore the mechanism by which RAGE polymorphisms regulate sRAGE expression. Future prospective cohort studies with large sample sizes are necessary to clarify the cause-effect relationships between serum sRAGE levels and DR.

## Figures and Tables

**Table 1:** Clinical characteristics of DR and NDR groups.

	DR (*n* = 372)	NDR (*n* = 668)	Statistic value
Gender (male/female)	146/226	242/426	0.334
Age (mean ± SD)	63.39 ± 10.60	62.58 ± 11.65	0.257
Diabetes duration	9.27 ± 6.78	5.56 ± 5.58	<0.001*
BMI (kg/m^2^)	24.43 ± 3.47	24.54 ± 3.86	0.635
WHR	0.92 ± 0.14	0.91 ± 0.07	0.158
Systolic blood pressure (mmHg)	133.85 ± 16.48	132.08 ± 15.32	0.091
Diastolic blood pressure (mmHg)	80.62 ± 10.46	79.57 ± 9.13	0.116
FPG (mmol/L)	8.47 ± 3.43	7.54 ± 3.19	<0.001*
2hPG (mmol/L)	13.01 ± 4.36	12.01 ± 4.30	<0.001*
HbA1c (%)	9.43 ± 2.33	8.65 ± 2.36	0.049*
Total cholesterol (mmol/L)	5.49 ± 1.02	5.43 ± 0.96	0.341
Triglycerides (mmol/L)	2.21 ± 1.27	2.13 ± 1.03	0.254
HDL-C (mmol/L)	1.30 ± 0.31	1.32 ± 0.32	0.297
LDL-C (mmol/L)	2.81 ± 0.46	2.91 ± 0.45	0.001*
sRAGE (pg/mL)	526.17 (381.96–798.33)	522.15 (360.79–739.80)	0.168

*Significant results.

Numbers are given as *n* (%).

**Table 2:** Genotype and allele frequencies of *RAGE* gene polymorphisms in diabetic patients with and without retinopathy.

Polymorphism	Genotype	Genotype distribution				Alleles	Allele frequency		
		Cases	Control	OR (95% CI)	*P *		Cases	Control	*P *
2184A/G rs3134940 (*n* = 982)	A/A	235	448	1.00	0.35	A	0.82	0.84	0.28
	A/G	108	167	0.81 (0.61–1.08)		G	0.18	0.16	
	G/G	8	16	1.05 (0.44–2.49)					

1704G/T rs184003 (*n* = 1039)	G/G	266	490	1.00	0.40		0.85	0.85	0.77
	G/T	98	156	0.86 (0.64–1.16)			0.15	0.15	
	T/T	8	21	1.42 (0.62–3.26)					

rs1035798 (*n* = 1038)	C/C	274	467	1.00	0.08	C	0.85	0.84	0.57
	C/T	83	183	1.29 (0.96–1.74)		T	0.15	0.16	
	T/T	15	16	0.63 (0.30–1.29)					

G82S rs2070600 (*n* = 1039)	G/G	195	396	1.00	0.0035*	G	0.70	0.76	0.0015*
	G/S	131	227	1.17 (0.89–1.54)		S	0.30	0.24	
	S/S	46	44	2.12 (1.36–3.32)					

−429T/C rs1800625 (*n* = 1037)	T/T	280	502	1.00	0.42	T	0.86	0.87	0.63
	C/T	80	150	1.05 (0.77–1.42)		C	0.14	0.13	
	C/C	12	13	0.60 (0.27–1.34)					

The Chi-square test was used to compare the genotype and allele frequencies between DR and NDR groups.

*P < 0.05 was considered statistically significant.

**Table 3:** Multilocus genetic interaction model.

Model	Training balance accuracy	Testing balance accuracy	CVC	OR 95% CI	*P* value
G82S	0.5353	0.5238	9/10	1.3297 (1.03, 1.7163)	0.0286
2184A/G, G82S	0.5565	0.5232	6/10	1.5972 (1.2277, 2.078)	0.0005
2184A/G, G82S, −429T/C	0.5732	0.5187	4/10	2.1518 (1.5975, 2.8983)	<0.0001
2184A/G, 1704G/T, G82S, −429T/C	0.5957	0.5492	10/10	2.2254 (1.7115, 2.8935)	<0.0001

The best model has the maximum CVC and highest testing balance accuracy.
